# ﻿*Ctenophorahunanensis* sp. nov. (Bacillariophyta) from the Yongle River in Hunan Province, China

**DOI:** 10.3897/phytokeys.250.140576

**Published:** 2024-12-27

**Authors:** Dong-Qin Xiang, Rui Yang, Patrick Rioual, Yan Zheng, Bing Liu

**Affiliations:** 1 College of Biology and Environmental Sciences, Jishou University, Jishou 416000, China Jishou University Jishou China; 2 Key Laboratory of Cenozoic Geology and Environment, Institute of Geology and Geophysics, Chinese Academy of Sciences, P.O. box 9825, Beijing 100029, China Institute of Geology and Geophysics, Chinese Academy of Sciences Beijing China; 3 CAS Center for Excellence in Life and Paleoenvironment, Beijing 100044, China CAS Center for Excellence in Life and Paleoenvironment Beijing China

**Keywords:** abnormal valve, central area, cribrum, *Ctenophora*, sternum

## Abstract

A new species, *Ctenophorahunanensis* Bing Liu & Rioual, **sp. nov.**, found in the Yongle River, a tributary of the Xiang River (Hunan Province, southern China) is described on the basis of morphological observations made under light and scanning electron microscopes. *Ctenophorahunanensis* is distinguished from other *Ctenophora* taxa by a unique combination of characters that includes its lanceolate valve outline with rostrate apices, sternum gradually becoming wider from valve apices to center, and a greater valve width than the other members of the genus. *Ctenophorahunanensis* inhabits the epilithic community in the headwaters of a freshwater river. Many abnormal valves of *C.hunanensis* were observed in the samples investigated and the most frequent morphological abnormalities consisted in a lack of symmetry relative to the apical axis caused by a unilateral incising in the middle part of the valve.

## ﻿Introduction

**Figure 1.**
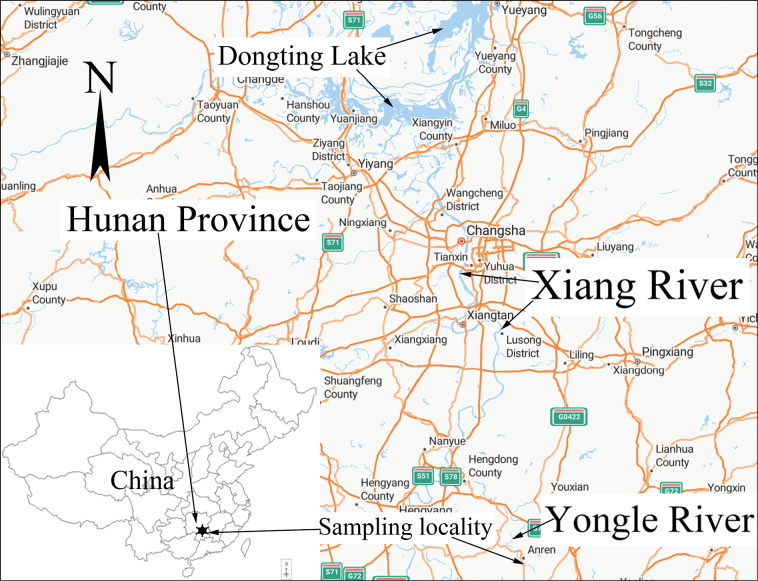
Map of Hunan Province, in southern China, showing the location of the Yongle River, a tributary of the Xiang River and Dongting Lake. The Yongle River is the type locality of *Ctenophorahunanensis* sp. nov.

In recent years, the diatom flora of Hunan Province in southern China (Fig. 1) has been investigated by Dr. Liu from Jishou University and his collaborators and their research led to the descriptions of several species new-to-science (e.g. Liu et al. 2016, 2017a, 2017b, 2017c, 2018a, 2018b, 2018c, 2019a, 2019b, 2019c, 2020a, 2021; Long et al. 2021, 2022a, 2022b; Liu 2023; Yuan et al. 2023; Xu et al. 2024). Many of these new species were described from Dongting Lake, the second largest freshwater lake in China that is located in the northeast of Hunan and drains the entire river system of this province with only a few exceptions. By contrast, the diatom flora of the rivers of Hunan, such as the Xiang River and its tributaries, has been relatively underexplored until now with only a few reports available in the literature (Long et al. 2022c, Liu 2023, Yuan et al. 2023, Zheng et al. 2023). Thus, as part of an ongoing investigation on the Xiang River and its tributaries, epilithon samples were collected from the Yongle River (Fig. 1) and analysed. The analyses revealed the presence of specimens of the genus *Ctenophora* (Grunow) D.M. Williams & Round that could not be attributed to any known species from this genus.

*Ctenophora* was raised to the generic level by Williams and Round (1986). As a genus, it is distinguished from other related genera by the type of valve striation (with transverse, uniseriate striae composed of poroids closed externally by complex cribra), the cribrum structure and the robust plain area (i.e. without perforations in the silica wall) that usually occupies the entire width of the valve in its center. For more than three decades, *Ctenophora* was thought to be monotypic, only represented by the species *Ctenophorapulchella* (Kützing) Williams & Round, which was originally described from England but considered as cosmopolitan. This situation changed with the discovery of *Ctenophorasinensis* Bing Liu & D.M. Williams (Liu et al. 2020b) from samples collected in Lake Qinghai (China). More recently, Williams and Van de Vijver (2023a, 2023b) re-examined type material from European historical collections and proposed the re-combination and transfer to *Ctenophora* of three species. An exhaustive discussion on the structural characters useful for the description of *Ctenophora* taxa and on the phylogenetic relationships of this genus with other genera such as *Tabularia* (Kützing) Williams & Round and *Catacombas* Williams & Round was also proposed in Williams and Van de Vijver (2023b).

This paper describes *Ctenophorahunanensis* Bing Liu & Rioual sp. nov., as a new freshwater species from China and further contributes to the under-appreciated diversity of *Ctenophora* as well as to the investigation of the diatom flora of Hunan Province.

## ﻿Materials and methods

The diatom samples of this study were collected from the Yongle River which runs through Anren County in the south of Hunan Province (Fig. 1). The Yongle River is a headwater tributary of the Xiang River which is the largest river in Hunan. Benthic diatom samples were collected on March 24, 2024. The method of collecting the diatom samples is the same as in Liu (2023) and consists of sampling numerous submerged stones showing yellow-brown surfaces that indicate the presence of diatoms. Immediately after being retrieved from the river bed, each stone was placed on a plastic plate and its surface was brushed using a toothbrush, with the brushed-off diatom samples being washed into the plate. The diatom samples were transferred into two 100 ml sampling bottles. One bottle was fixed with 70% ethanol and the other was left unfixed. At the time of sample collection, water temperature, pH, and conductivity were measured in situ with a portable multimeter (HQ40D, Hach Company, USA).

The laboratory methods are also the same as in Liu (2023) and consist as follows: “The collected diatom samples which were added 70% alcohol were processed (cleaned) for microscopic examination with 10% HCl and 30% H_2_O_2_. Permanent slides were prepared using Naphrax mountant and examined using a Leica DM3000 light microscope (LM). Slides are deposited in the Herbarium of Jishou University, Hunan, People’s Republic of China (**JIU**) (Herbarium acronyms follow Index Herbarium http://sweetgum.nybg.org/science/ih/). Samples were further examined using a field emission scanning electron microscope (SEM, Carl Zeiss Microscope, model Sigma HD) available at Huaihua University, China. For SEM analysis, several drops of the cleaned diatom material were air-dried onto glass coverslips. The coverslips were attached to aluminum stubs using double-sided conductive carbon strip and sputter-coated with platinum (Cressington Sputter Coater 108auto, Ted Pella, Inc.). The terminology used in the description and in the discussion mainly follows Round et al. (1990) and Liu et al. (2020b).

## ﻿Results

### ﻿ Ctenophora hunanensis

Taxon classificationPlantaeDipteraTipulidae

Bing Liu & Rioual sp. nov.

2F3135BC-BBD4-5344-A321-1A9BB1829F96

Figs 2 , 3 , 4 , 5

#### Holotype.

**Figure 2.**
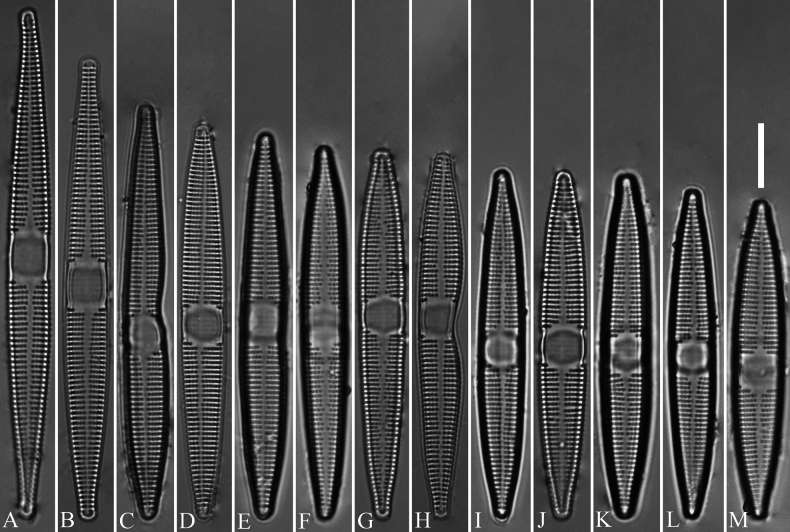
*Ctenophorahunanensis* sp. nov., LM × 1000 **A–M** thirteen valves showing a size diminution series, note three abnormal valves (**C, E, H**) **B** illustration of holotype specimen. Scale bar: 10 μm (**M**).

Specimen circled on slide DIA2024010 (= Fig. 2B), deposited in the Herbarium of Jishou University (JIU), China. Registration: http://phycobank.org/105130.

#### Type locality.

China. Hunan Province: Anren County, Yongle River, sampling site with the coordinates 26°50'12"N, 113°35'59"E, and an elevation of 123 m asl. Diatom samples collected by Bing Liu, March 24, 2024.

#### Description.

***LM*** (Fig. 2). Valves lanceolate with rostrate apices. Valve dimensions (n = 30): length 48–78 μm, width 6.5–8.5 μm at center. Sternum clearly visible, gradually widening when approaching central area. Central area distinct, rectangular to square, sometimes with ghost striae. Striae parallel, uniseriate, perpendicular to central sternum, opposite one another across sternum. Stria density 13–16 in 10 μm. Areolae punctate, 20 in 10 μm. Many abnormal valves are found, all of which exhibit the asymmetry relative to the apical axis due to the V-shaped incising of valve margin only occurring at one side (Fig. 2C, E, H).

**Figure 3.**
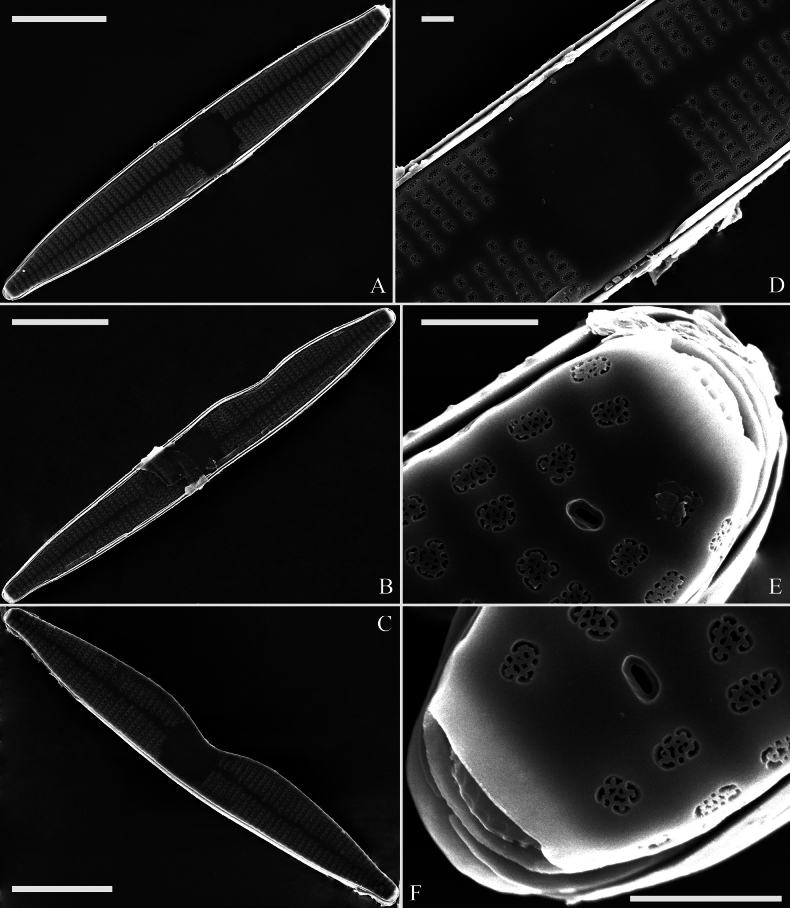
*Ctenophorahunanensis* sp. nov., external view, SEM**A** normal frustule **B, C** two abnormal frustules **D** middle part, detail from **A** note the central area and sternum **E, F** details of the two apices of the valve shown in **A** note the complex cribrae, the external openings of the rimoportulae (one on each apex), and the ocellulimbi. Scale bars: 10 μm (**A–C**); 1 μm (**D–F**).

**Figure 4.**
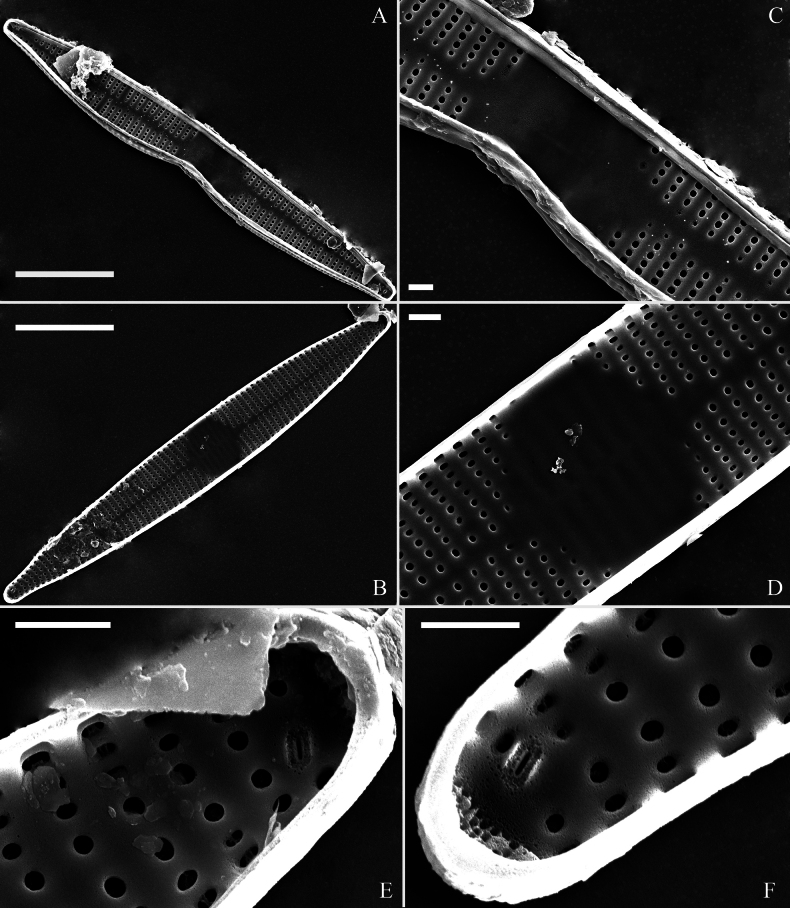
*Ctenophorahunanensis* sp. nov., internal view, SEM**A** abnormal valve **B** normal valve **C** middle part, detail from **A** showing the central area **D** middle part, detail from **B** showing the central area **E, F** details of the apices from **B**. Scale bars: 10 μm (**A, B**); 1 μm (**C–F**).

**Figure 5.**
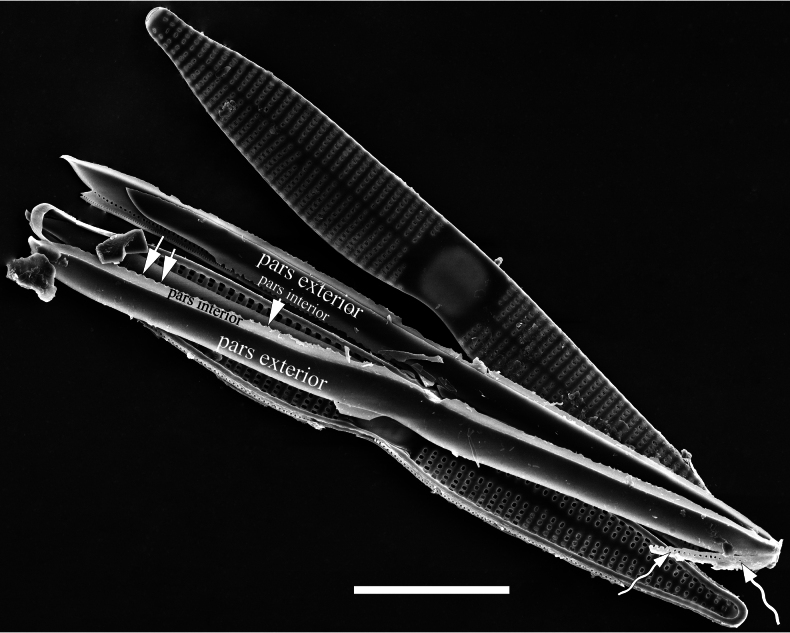
*Ctenophorahunanensis* sp. nov., SEM A dissembled frustule, note the distinctive central area, open, unornamented valvocopula, on its advalvar edge, valvocopula bears a row of serrated projections, each corresponding internally to a virga (three white arrows). Copulae with row of poroids situated at pars media (two white wavy arrows). Scale bar: 10 μm.

***SEM*** (Figs 3–5). Sternum meeting and integrated with virgae, vimines reduced in size relative to virgae. Virgae and vimines join to form square to rectangular areolae that are closed externally by complex cribra appearing as sieve-like closing plates composed of pegged struts (Fig. 3D–F). Inner openings of areolae appear rounded (Fig. 4C, D). Central area distinctive, externally a broad plain area (Fig. 3D), internally thickened around its periphery, ghost striae within (Fig. 4D). One rimoportula present at each apex, externally expressed as a simple hole (Fig. 3E, F), internally bilabiate, situated close to sternum (Fig. 4E, F). Ocellulimbus produced at each apex, typically sunken below surface of valve margin (Fig. 3E, F). Valvocopula open, lacking ornamentation, distinctly deeper than copulae (Fig. 5). On its advalvar edge, valvocopula bears a row of serrated projections, each corresponding internally to a virga (Fig. 5, three arrows). Copulae with row of poroids situated at pars media (Fig. 5, two wavy arrows).

#### Etymology.

Named after Hunan Province where the species was found.

#### Distribution and ecology.

Known only from the type locality. The samples that included this species were scraped off the surface of stones collected in the Yongle River. Hence this is a benthic, epilithic species. The relative abundance of this new species is ca. 3%. The associated taxa include *Gomphonemaparvulum* (Kützing) Kützing, *Luticola* spp., *Planothidiumfrequentissimum* (Lange-Bertalot) Lange-Bertalot, *Tryblionella* spp., *Nitzschia* spp., among others. The following environmental parameters were measured in the field with three replications: Conductivity = 85.9 ± 0.1 μS cm^-1^; pH = 8.9 ± 0.1; Water temperature = 22.3 ± 0.3 °C.

## ﻿Discussion

The general morphology of *Ctenophorahunanensis* Bing Liu & Rioual sp. nov. has all the characteristic features of the genus *Ctenophora*, including transverse, uniseriate striae composed of poroids closed externally by complex cribra, and the robust plain area that usually occupies the entire width of the valve in its center. Currently in AlgaeBase (Guiry in Guiry and Guiry 2023) for the genus *Ctenophora* there are five names taxonomically accepted as species. The five species include *C.pulchella*, the type species of the genus, *C.saxonica* (Kützing) D.M. Williams & Van de Vijver, *C.sinensis* B. Liu & D.M. Williams, *C.subula* (Sande Lacoste & Suringar) D.M. Williams & Van de Vijver, and *C.vertebra* (W. Gregory) D.M. Williams & Van de Vijver. Compared to the five other species of *Ctenophora* listed above, *C.hunanensis* is distinguished by its valve outline (lanceolate with rostrate apices), a sternum gradually becoming wider from the valve poles to center, and a much greater valve width than the other *Ctenophora* taxa (Table 1). *Ctenophorasinensis* and *C.vertebra* have very different valve outlines and can therefore be easily distinguished from *C.hunanensis*. *Ctenophorahunanensis* could be possibly confused with the smaller valves of *C.pulchella* and *C.saxonica* (and possibly *C.subula*?), but the difference in valve width remains (i.e., the ranges in width do not overlap). In addition, the central area of *C.hunanensis* is not buttressed while those of *C.pulchella* and *C.subula* are strongly buttressed.

**Table 1.** Comparisons between *Ctenophorahunanensis* sp. nov. and the other species of the genus *Ctenophora*.

Feature	*C.hunanensis* sp. nov.	*C.pulchell*a	*C.saxonica*	*C.sinensis*	*C.subula*	*C.vertebra*
Valve outline	Lanceolate with rostrate apices	Lanceolate with sub-capitate apices	Lanceolate with sub-capitate apices	Lanceolate with capitate to sub-capitate apices	Lanceolate, tapering towards the poles	Lanceolate with capitate apices
Valve length (L) and width (W) (μm)	L: 48–78; W: 6.5–8.5	L: ca. 48–59; W: ca. 2–4	L: ca. 51–97; W: ca. 4–6	L: 70–136; W: 4–6	L: 65–80; W: 2–5	L: ca. 44–104 μm; W: 2–4 μm
Striae in 10 μm	13–16	13–14	13–14	15–19	13–14	No data
Areolae in 10 μm	20	ca. 20	ca. 20	26–28	ca. 20	No data
Cribrum	Sieve-like closing plates	Mesh-work with ca. 4–8 strutted closing plates	Mesh-work with ca. 6–8 closing plates	Sieve-like closing plates	Mesh-work with ca. 8–12 strutted closing plates	No data
Sternum	Gradually widening from pole to center	Gradually widening from pole to center	Very narrow, linear	Very narrow, linear	Relatively narrow, linear	Very narrow, linear
Central area	Square to rectangular, not butressed	Square to broadly circular-oblong, butressed	Square to broadly circular-oblong, not butressed	Rectangular to square, weakly butressed	Square to oblong, heavily butressed	Ovoid
Habitat	Freshwater	Freshwater to brackish	Fresh to salty water	Brackish water	Freshwater	Freshwater (?)
References	This study	Williams and Van de Vijver (2023b)	Williams and Van de Vijver (2023b)	Liu et al. (2020b)	Williams and Van de Vijver (2023a)	Williams and Van de Vijver (2023b)

Regarding the ultrastructure of the cribra, *C.hunanensis* possesses sieve-like closing plates composed of pegged struts that appear similar to those of *C.sinensis*, the other species described from China. In the three species described from Europe for which the structure has been illustrated, i.e. *C.pulchella*, *C.saxonica*, *C.subula*, the cribra appear as mesh-like structures. This difference in cribra structure may however be an artifact of the valve preservation, as the specimens illustrated by Williams and Van de Vijver (2023a, 2023b) were taken from historical collection at least 160 years old and may have been eroded to some extent.

Until recently as the genus was thought to be monotypic, *Ctenophora* was generally considered as an indicator of high electrolyte content in brackish waters, in marine coasts and river estuaries. When found in inland waters, *Ctenophora* was encountered in saline springs and salinized running waters. Its presence in freshwater was possible, but only in low numbers. It was also considered tolerant of polluted water, up to the α-mesosaprobic level (Lange-Bertalot et al. 2017). The discovery (or re-discovery) of other species of *Ctenophora* already suggested that populations of this genus could live in freshwater habitats. For example, Williams and Van de Vijver (2023a) reported that *Ctenophorasubula* was found in the Netherlands as epiphytic on the filamentous green alga “*Cladophora Sandii*” (syn.: *Cladophorarivularis* (L.) Kuntze), which is a freshwater species. The salinity preference of *Ctenophoravertebra* is difficult to assess precisely although the type location “lacustrine sands, Glenshira, near Inveraray” in Scotland, also suggests it comes from a freshwater habitat (Williams and Van de Vijver, 2023b). The discovery of *C.hunanensis* in the headwaters of the Yongle River therefore confirms that the ecological niche of the genus *Ctenophora* is very broad, not just restricted to brackish conditions but also includes low-conductivity, unpolluted freshwater habitat.

Interestingly, the type population of *C.hunanensis* includes many abnormal valves (e.g., Figs 2C, E, H; 3B, C; 4A) which in the samples we investigated represented 23% of the specimens observed (7 abnormal valves out of 31). Most abnormal valves of *C.hunanensis* exhibit abnormal valve outlines lacking symmetry relative to the apical axis due to the incising of valve margin only occurring at one side (Figs 2C, E, H, 3B, C, 4A), which is a type of abnormality commonly reported in araphid diatoms (Falasco et al. 2009, 2021).

These observations further illustrate the morphological plasticity reported for some araphid genera such as *Hannaea*, *Ulnaria* and *Fragilaria*. For *Ulnaria* in particular, Zheng et al. (2024) also reported a large percentage of abnormal valves, with an asymmetrical incision on one margin of the valve, from the type population of *Ulnariashun-biseriata* Bing Liu & Rioual, which was also described as a new species from a river in Hunan province. Further research is required to explain why diatom populations with large proportions of abnormal valves appear to be common in rivers of Hunan.

## Supplementary Material

XML Treatment for Ctenophora hunanensis

## ﻿Additional information

### Author ORCIDs

Dong-Qin Xiang https://orcid.org/0009-0009-5498-9479

Rui Yang https://orcid.org/0009-0009-1150-6641

Patrick Rioual https://orcid.org/0000-0001-9491-9197

Yan Zheng https://orcid.org/0000-0001-7074-0678

Bing Liu https://orcid.org/0000-0002-8516-325X

### Data availability

All of the data that support the findings of this study are available in the main text.
